# Bone Structure Analysis of the Radius Using Ultrahigh Field (7T) MRI: Relevance of Technical Parameters and Comparison with 3T MRI and Radiography

**DOI:** 10.3390/diagnostics11010110

**Published:** 2021-01-12

**Authors:** Mohamed Jarraya, Rafael Heiss, Jeffrey Duryea, Armin M. Nagel, John A. Lynch, Ali Guermazi, Marc-André Weber, Andreas Arkudas, Raymund E. Horch, Michael Uder, Frank W. Roemer

**Affiliations:** 1 Department of Radiology, Massachusetts General Hospital, Harvard University, Boston, MA 02114, USA; 2 Department of Radiology, Friedrich Alexander University Erlangen-Nürnberg (FAU) & Universitätsklinikum Erlangen, 91054 Erlangen, Germany; Rafael.heiss@uk-erlangen.de (R.H.); armin.nagel@uk-erlangen.de (A.M.N.); michael.uder@uk-erlangen.de (M.U.); frank.roemer@uk-erlangen.de (F.W.R.); 3 Department of Radiology, Brigham and Women’s Hospital, Harvard University, Boston, MA 02114, USA; jduryea@bwh.harvard.edu; 4 Medical Physics in Radiology, German Cancer Research Center (DKFZ), 69120 Heidelberg, Germany; 5 Department of Epidemiology and Biostatistics, University of California San Francisco (UCSF), San Francisco, CA 94143, USA; JLynch@psg.ucsf.edu; 6 Department of Radiology, Boston University School of Medicine, Boston, MA 02118, USA; guermazi@bu.edu; 7 Department of Radiology, Boston Veteran Affairs Healthcare System, West Roxbury, MA 02132, USA; 8 Institute of Diagnostic and Interventional Radiology, Pediatric Radiology and Neuroradiology, University Medical Center Rostock, D-18057 Rostock, Germany; marc-andre.weber@med.uni-rostock.de; 9 Department of Plastic and Hand Surgery, Friedrich Alexander University Erlangen-Nürnberg (FAU) & Universitätsklinikum Erlangen, 91054 Erlangen, Germany; andreas.arkudas@uk-erlangen.de (A.A.); Raymund.Horch@uk-erlangen.de (R.E.H.)

**Keywords:** bone structure, wrist, MRI, X-ray, fractal signature analysis

## Abstract

Bone fractal signature analysis (FSA—also termed bone texture analysis) is a tool that assesses structural changes that may relate to clinical outcomes and functions. Our aim was to compare bone texture analysis of the distal radius in patients and volunteers using radiography and 3T and 7T magnetic resonance imaging (MRI)—a patient group (*n* = 25) and a volunteer group (*n* = 25) were included. Participants in the patient group had a history of chronic wrist pain with suspected or confirmed osteoarthritis and/or ligament instability. All participants had 3T and 7T MRI including T1-weighted turbo spin echo (TSE) sequences. The 7T MRI examination included an additional high-resolution (HR) T1 TSE sequence. Radiographs of the wrist were acquired for the patient group. When comparing patients and volunteers (unadjusted for gender and age), we found a statistically significant difference of horizontal and vertical fractal dimensions (FDs) using 7T T1 TSE-HR images in low-resolution mode (horizontal: *p* = 0.04, vertical: *p* = 0.01). When comparing radiography to the different MRI sequences, we found a statistically significant difference for low- and high-resolution horizontal FDs between radiography and 3T T1 TSE and 7T T1 TSE-HR. Vertical FDs were significantly different only between radiographs and 3T T1 TSE in the high-resolution mode; FSA measures obtained from 3T and 7T MRI are highly dependent on the sequence and reconstruction resolution used, and thus are not easily comparable between MRI systems and applied sequences.

## 1. Introduction

Magnetic resonance imaging (MRI)-based texture analysis has been used on different organs, including brain, breast and prostate [1,2,3]. For instance, it may provide a tool as noninvasive prognostic and predictive biomarkers—for example, in breast [3] and prostate [2]. In bone, fractal signature analysis (FSA) is a computerized method for the textural analysis of cancellous bone commonly extracted from plain radiographs which quantifies trabecular organization, taking into account the trabecular number, spacing and cross-connectivity [4,5]. Fractal dimension (FD), a parameter calculated using FSA, has been reported to predict structural outcomes such as radiographic osteoarthritis (OA) progression [6,7] and also clinical endpoints such as total joint replacement when measured in the knee [8]. FDs obtained from the distal radius in patients with rheumatoid arthritis were reported to correlate with longitudinal bone loss, particularly in those with higher grade radiographic severity [9]. Other studies of bone texture of the distal radius have reported structural information associated with bone mineral density [10,11]. On the other hand, data on FSA of the wrist in OA or pre-OA remain sparse [12,13].

The use of magnetic resonance imaging (MRI) for the study of distal radius cancellous bone texture was first reported more than two decades ago [14,15]. MRI provides high contrast between the hyperintense signal of the fatty marrow and hypointense-appearing trabecular bone plates and rods, and has been used for cancellous bone microarchitecture on standard 1.5 Tesla (T) or 3T platforms [16,17]. MRI-extracted FSA has the potential to be applied to a larger clinical population as a prognostic tool, especially since standard and widely available MRI clinical sequences are used for data extraction. However, its applicability in a routine clinical environment needs to be further explored. 7T ultrahigh field MRI of the wrist may provide improved diagnostic accuracy to determine the degree and location of structural damage [18,19], potentially helping in treatment decisions for further management (conservative versus surgical, as well as type of surgery). Given its high tissue contrast and superior spatial resolution, 7T MRI may also play a contributing role in the study of bone texture analysis of the distal radius. However, to date, the comparability of 7T extracted bone structure parameters with more standard measures such as 3T MRI or radiography has not been established. The role of MRI-depicted trabecular bone texture of the wrist as a potentially predictive instrument of treatment outcomes or functional performance is not well understood.

We hypothesize that cancellous bone structure analysis may be performed with a shorter acquisition time on standard 7T wrist MRI, yielding comparable results to standard 3T MRI using different degrees of reconstructed resolution and to FSA parameters extracted from plain radiographs. As the increased signal-to-noise ratio (SNR) available at 7T MRI allows for ultra-high-resolution imaging, we hypothesize further that a high-resolution (HR) T1-weighted (w) sequence at 7T may yield more comparable results compared to X-rays in contrast to a standard T1w sequence at 7T. 

The aim of this study is to show the feasibility of distal radius FSA extracted from a clinical 7T MRI system using a standard and a HR T1w sequence with different levels of reconstructed resolution and compare the 7T MRI-derived FSA parameters to FSA parameters obtained from 3T MRI and radiography.

## 2. Methods

### 2.1. Study Cohort

The study design was approved by the local institutional review board of Friedrich-Alexander University, Erlangen-Nürnberg (163_13 B) and University Medical Center Rostock (A 2018-0126). Fifty subjects chosen via convenience sample were enrolled in this study, including a patient group (*n* = 25) and a healthy volunteer group (*n* = 25). The patient group included participants with chronic wrist pain that were referred for outpatient consultation at a tertiary referral center for hand surgery with suspected or confirmed OA and/or ligament instability. The exclusion criteria were a history of trauma within the last 6 months, a history of inflammatory arthritis and inability to undergo 3T or 7T MRI. Of note, no participant had a history of prior distal radial fracture.

### 2.2. Radiography

Twenty-three participants from the patient group had a frontal radiograph of the wrist. Images were acquired in a digital fashion at a film-focus distance of 105 cm. All radiographic images were reviewed by a musculoskeletal radiologist (MJ) for quality assurance, which was based on optimal visualization of the radiocarpal joint space. All 23 radiographs were deemed satisfactory for evaluation.

### 2.3. Magnetic Resonance Imaging

All participants in the patient and volunteer groups had both 3T and 7T MRI examinations of the wrist. Examinations were performed on clinical 3T (Siemens Magnetom Vida, Erlangen, Germany) and 7T systems (Siemens Magnetom Terra, Erlangen, Germany). For 3T MRI, a 16-channel receive hand-wrist radiofrequency coil (Siemens, Erlangen, Germany) was used. For 7T MRI, a prototype 1-channel transmit/16-channel receive wrist radiofrequency coil (Rapid Biomedical GmbH, Rimpar, Germany) was employed. The 3T examination included a T1w turbo spin echo (TSE) sequence acquired in all participants, both in the patient and volunteer groups. 7T examinations included two different coronal T1w TSE sequences: 7T T1w TSE-fast and 7T T1 TSE-HR. MR imaging parameters for each sequence are presented in Table 1. 7T T1w TSE-fast images were not available for 11 participants of the volunteer group and for 3 participants of the patient group. 7T T1 TSE-HR images were available in all participants from both groups. MRI were reviewed for quality assurance (MJ) and deemed satisfactory for evaluation. MRI with minimal motion artifacts that would have been otherwise acceptable for clinical assessment were included. In total, 7 examinations showed minimal motion artifacts.

### 2.4. Bone Texture Analysis

**Figure 1:**
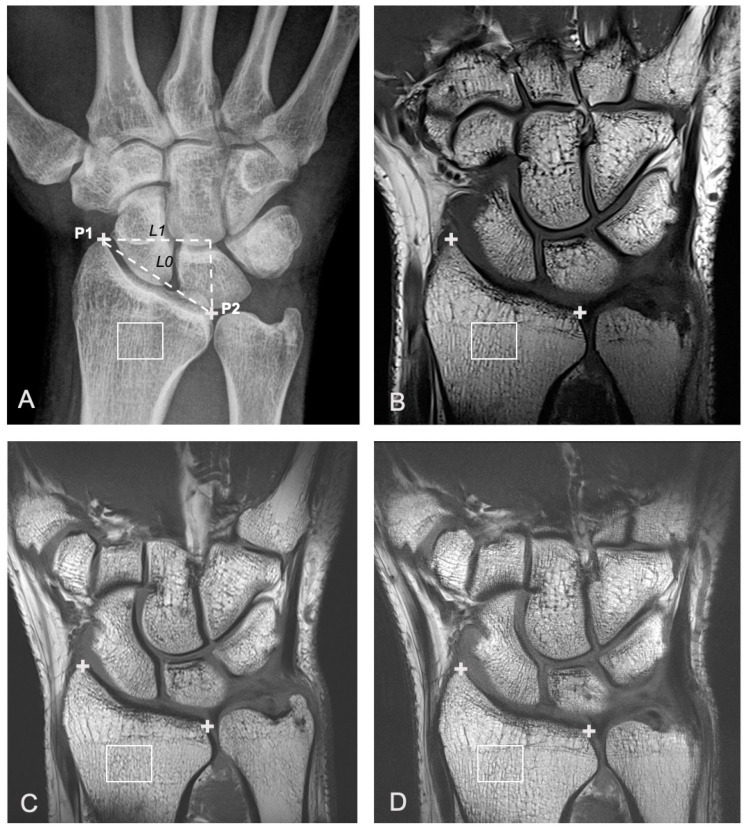
A 31-year-old female with ulnar-sided right wrist pain that had lasted several months. Right wrist imaged with different imaging modalities in a participant within the patient group, including frontal a.p. radiographic view (**A**), 3T coronal T1w turbo spin echo (TSE) MR image (**B**), and two different 7T coronal T1w TSE MR images acquired with different TE and TR parameters (**C**: 7T T1w TSE-fast and **D**: 7T T1 TSE-high resolution (HR)). The center of the region of interest (ROI) (white box) is located 0.4 × L1 on the ulnar side of P1 (+) (in the horizontal direction) and 0.25 × L0 below P2 (+) (**A**–**D**). The width and height of the ROI were 0.4 × L0 and 0.3 × L0, respectively. L0 is defined as the distance between P1 and P2 (oblique dashed line). L1 is defined as the horizontal distance between P1 and P2 (horizontal dashed line) (**A**).

Predefined regions of interest (ROIs) were created in the distal radius on a single coronal slice, based on two landmarks, P1 and P2, placed manually by a musculoskeletal radiologist with 10-year experience in MRI assessment of OA (MJ). The selected slice corresponds to the central slice between the anterior and posterior radial cortices. The landmarks were positioned along the radial and ulnar aspects of the distal radius. P1 corresponds to the most distal aspect of the radius along the radial side. P2 corresponds to the most distal aspect of the radius along the ulnar side. The distance measured between P1 and P2 is referred to as L0. The distance between two vertical lines passing though P1 and P2, respectively, is referred to as L1. The ROI was centered at a point 0.4 × L1 from P1 in the horizontal direction and 0.25 × L0 below P2 in the vertical direction. The width and height of the ROI were 0.4 × L0 and 0.3 × L0, respectively (Figure 1). Pixel spacing was 0.148 × 0.148 mm for radiography. The voxel spacing was 0.2 × 0.2 × 2 mm for 3T T1 TSE and 7T T1 TSE-fast MRI. The voxel spacing was 0.125 × 0.125 × 2 mm for 7T T1 TSE HR MRI. FSAs were calculated in the horizontal and vertical dimensions according to a modified Buckland-Wright method [5]. The modification was required to account for the differing image pixel sizes from X-ray (0.148 mm) and MRI (0.124 or 0.2 mm). This was performed by estimating fractal dimensions for horizontal and vertical directions over 3 different resolution ranges (high resolution: 0–0.9 mm, medium resolution: 0.9–1.8 mm and low resolution 1.8–2.7 mm).

### 2.5. Analytic Approach

Two sample t-test was performed to compare the horizontal and vertical FDs between the patient group and volunteer group for each available MRI sequence and for each resolution mode. A paired t-test was performed to determine whether there is a difference in horizontal and vertical FDs between radiography and all of the 3T and 7T T1 TSE MRI sequences. In all statistical tests, an effect was considered to be statistically significant if the *p*-value was less than 0.05. All statistical analyses were performed in SAS, version 9.4 (SAS Institute, Inc., Cary, NC, USA).

## 3. Results 

Fifty participants were included. The patient group (*n* = 25) included 11 females and 14 males, with a mean age of 39.0 (range: 18–76). The volunteer group (*n* = 25) included 16 females and 9 males, with a mean age of 25.1 (age range: 16–34). A detailed overview of the results for the horizontal and vertical FDs for both the patient and volunteer groups is presented in Table 2 and Table 3. In summary, for both the horizontal and vertical FDs a significant difference between the patient and volunteer groups was observed for the low-resolution mode extracted from 7T T1w TSE-HR images (*p*-values = 0.04 for horizontal and 0.01 for vertical). 

A comparison of mean FDs between radiography and 3T T1w TSE, 7T T1w TSE-fast and 7T T1w TSE-HR is presented in Table 4 and Table 5. When using radiography as a reference, we found a statistically significant difference of horizontal FDs with 3T T1w TSE in high-resolution mode, and with 7T T1w TSE-HR for low- and high-resolution modes. For vertical FDs, we found statistically significant differences between radiography and 3T T1 TSE for the low-resolution mode.

**Figure 2:**
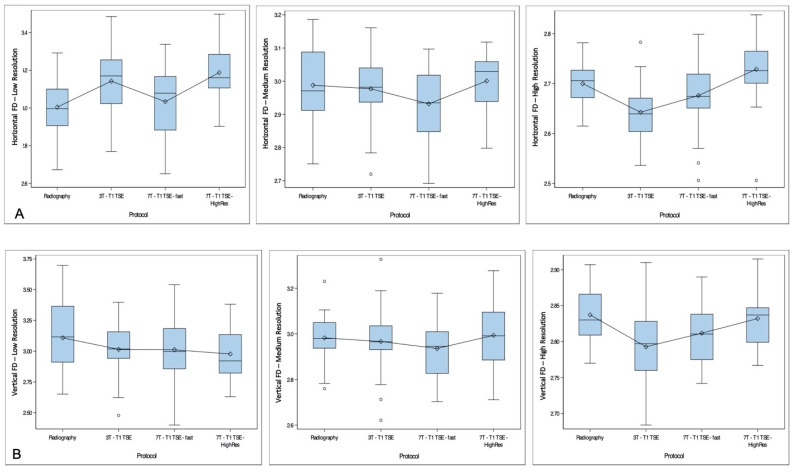
Curve fitting of horizontal (**A**) and vertical (**B**) fractal dimensions (FDs) by modality and according to resolution mode. Each modality is presented in the X-axis, with FD in the Y-axis. High-, medium- and low-resolution curves are presented separately. No consistent distribution of FDs is found between the different resolution modes. However, for the high-resolution mode, horizontal and vertical FDs extracted from 3T T1w STE images appear to be lower than FDs extracted from other imaging modalities. In medium resolution, FDs extracted from 7T T1w TSE-fast appear to be slightly lower than those extracted from other imaging modalities.

Curve fitting of horizontal and vertical FDs by modality and resolution mode showed no consistent distribution of horizontal and vertical FDs (Figure 2). In the high-resolution mode, horizontal and vertical FDs extracted from 3T T1w TSE images appeared to be lower in comparison with radiography and both 7T T1w TSE sequences (mean horizontal FD of 2.63 versus 2.68–2.73 for other modalities, and mean vertical FD of 2.79 versus 2.81–2.83 for other modalities). However, in medium-resolution mode, FDs extracted from 7T T1 TSE-fast appeared to be slightly lower than those extracted from other modalities (horizontal: 2.93 versus 2.98–3.0 for other modalities, and vertical: 2.94 versus 2.9–2.99 for other modalities).

## 4. Discussion

Our results seem to suggest that there is no consistent FD trend between radiography, 3T T1w TSE and 7T T1w TSE-fast and 7T T1w TSE-HR sequences. Our results suggest that a change in MRI acquisition and reconstruction parameters may significantly alter extracted trabecular bone FSA and, thus, absolute values need to be interpreted with caution. The observed differences between mean horizontal and vertical FDs and between radiography and different MRI sequences suggest a wide variability of bone texture results depending on the technique used. 

MRI is a potentially useful method for imaging of trabecular bone in vivo, first because of its noninvasiveness and lack of ionizing radiation, but also due to high contrast between bone marrow and trabecular bone, which appears as background intensity [17]. Analysis of trabecular bone is particularly suitable for the distal radius compared to structures located deeper in the body such as the hip joint. The close proximity to the radiofrequency coil provides a higher SNR, which, in turn, helps achieve lower voxel sizes. In addition, SNR gains can be facilitated by scanning at higher field strengths [20]. 7T MRI has a higher SNR, improved spatial resolution and allows for faster imaging compared to 1.5T and 3T MRI. For these reasons, 7T MRI has great potential for musculoskeletal applications in general and imaging of the wrist and the hand in particular. For instance, high-resolution 7T MRI could significantly improve diagnostic confidence and accuracy, allowing optimal visualization of small structures such as triangular fibrocartilage [18].

The variability of MRI-based quantitative measures depending on scanning parameters has been acknowledged previously in the context of compositional MRI techniques assessing cartilage ultrastructure [21] and seems similarly relevant for bone structure analysis. Prior studies have focused on the reproducibility of MRI-based bone microarchitecture assessment in the wrist [22,23,24]. Wald et al. showed that increased SNR may enhance reproducibility of structural and mechanical parameters derived from high-resolution MRI in the distal tibia, including the preferential direction of trabecular bone and trabecular thickness [23]. 

It had previously been reported that even subtle motion during the scan can cause large errors in the derived structural parameters in the distal radius [25,26]. Prior reports used tight immobilization of the anatomic region of interest [23], as well as further retrospective use of navigators [27] and autofocusing [26] to minimize motion corruption. In our study, quality assurance assessment allowed for minimal motion degradation, similar to the standard of diagnostic MRI. Our goal was to test the reproducibility of bone texture parameters in conditions most similar to clinical practice and trial settings.

The effect of MRI acquisition parameters was also extensively studied in the field of radiomics and texture analysis in general, using both MRI [28,29,30] and CT data [31,32,33]. Using MRI, Mayerhoefer et al. found that changes in repetition time (TR)/Echo time (TE), sampling bandwidth and number of acquisitions had a substantial impact on the sensitivity of the texture analysis features in phantoms [29]. In addition to these parameters, Buch et al. showed the effect of a number of excitation, flip angle, magnet strength, and scanner platforms on texture analysis results on a nonanatomic phantom [28]. The latter study, which used 1.5 and 3T scanners, suggested that a change in magnetic field may be a reason for variation in texture analysis in phantoms. Apart from MRI-based techniques, we have reported previously on radiographic bone texture analysis and found that geometric projection (anteroposterior versus posteroanterior projection) may have a marked impact on trabecular bone FSA [34]. Therefore, standardization and generalization of bone texture analysis are a major challenge to be accounted for, and overcome before MRI-based bone texture analysis, can be used on a larger scale and compared across sites and platforms.

By using 7T T1 TSE-HR in low-resolution mode, we demonstrated a statistically significant difference between horizontal and vertical FDs between the patient and volunteer groups. However, this finding does not appear to be reproducible across medium/high-resolution modes and for other tested MRI sequences. These findings also underscore the variability of bone texture results depending on MRI scanning parameters. Of note, a recent study showed that radiography-based texture analysis derived from previously published methods in the knee could not detect OA in the radiocarpal joint [12]. In our study, we were not able to compare radiography-based FSA results between both groups since the “volunteer” group did not undergo radiographs. 

Our results show the importance of understanding the effect of imaging acquisition parameters on FSA of the trabecular bone. While bone texture analysis is a potentially valuable analytic tool for characterizing the complicated trabecular structure of bone, it is crucial to distinguish bone texture analysis variations related to MRI technical acquisition and reconstruction parameters from changes secondary to pathology. Our study underscores the importance of a standardized and rigorously controlled scanning protocol when conducting research using bone texture analysis and particularly in longitudinal analyses. The evolution of compositional MRI of cartilage over the last two decades seems comparable to the next steps that need to be undertaken to make FSA a more clinically applicable instrument. Indeed, early studies in compositional MRI of cartilage showed suboptimal multivendor reproducibility [21]. This lack of reproducibility was later remediated by standardization efforts [35,36], including the Quantitative Imaging Biomarkers Alliance (QIBA) endorsed by the Radiologic Society of North America (RSNA) [37]. These efforts have placed compositional cartilage MRI as a potential tool for predicting functional outcomes by assessing change within an individual over time and particularly in monitoring the earliest disease stages that are likely to be the ones most amenable to nonsurgical therapy [38,39]. We believe similar efforts will be needed for bone texture analysis so that we can determine whether structural changes may potentially be related to functional outcomes.

There are several limitations to the current study. First, the studied sample is small, which limits the statistical power of our study. Due to the small size of this sample, we could not adjust for age and gender. In addition, our study does not account for minimal variations that may have occurred during MRI acquisition and ROI placement, nor does it account for minimal motion-related artifacts. However, such small variations are inherent to routine clinical protocols and would be difficult to control in a clinical practice. The continued development of new MRI technology and the continued improvement of MRI sequences will help identify robust FSA features that are reproducible across imaging sequences. Last, there are other bone texture analysis features that we did not include in our study including first and second order statistics features [40], and Gray Level Cooccurrence Matrix (also known as GLCM) computational analysis [41]. Future studies should also look at these parameters to determine their reproducibility across imaging sequences.

## 5. Conclusions

FSA measures obtained from 3T and 7T MRI appear to be highly dependent on sequence and imaging parameters and so are not easily comparable with MRI systems with different field strengths and applied sequences. Future work with more highly powered studies is needed to shed more light on this subject and evaluate the potential role of ultrahigh field MRI for structural analysis of bone.

## Figures and Tables

**Table 1:** MR imaging parameters for each sequence.

	Voxel Size (mm^3^)	Distance Factor (%)	Echo Time (TE) (ms)	Repetion Time (TR) (ms)	Flip Angle (Degrees)	Acceleration Factor	Field of View (FOV) Read (mm)	FOV Phase (%)	Time of Acquisition (s)	Number of Averages
**3T-T1 Turbo Spin Echo (TSE)**	0.2 × 0.2 × 2	10	15	600	90/150	2	90	100	269	3
**7T-T1 TSE–fast**	0.2 × 0.2 × 2	10	8.9	874	90/150	2	90	100	79	1
**7T-T1 TSE-HR**	0.125 × 0.125 × 2	10	13	1200	90/150	2	90	100.8	196	1

**Table 2:** Comparison of horizontal fractal dimensions between the patient group and volunteer group using 3T and 7T magnetic resonance imaging (MRI).

	Low Resolution (1.8–2.7 mm)			Medium Resolution (0.9–1.8 mm)			High Resolution (1.8–2.7 mm)		
	Patient	Volunteer	*p*-Value	Patient	Volunteer	*p*-Value	Patient	Volunteer	*p*-Value
**3T-T1 TSE**	3.142	3.128	0.77	2.977	2.947	0.34	2.629	2.657	0.06
**7T-T1 TSE–fast**	3.033	2.994	0.60	2.932	2.823	0.03	2.685	2.663	0.33
**7T-T1 TSE-HR**	3.188	3.047	0.04	3.001	2.967	0.37	2.731	2.727	0.79

**Table 3:** Comparison of vertical fractal dimensions between the patient group and volunteer group using 3T and 7T MRI.

	Low Resolution (1.8–2.7 mm)			Medium Resolution (0.9–1.8 mm)			High Resolution (0.0–0.9 mm)		
	Patient	Volunteer	*p*-Value	Patient	Volunteer	*p*-Value	Patient	Volunteer	*p*-Value
**3T-T1 TSE**	3.014	3.120	0.10	2.966	3.016	0.23	2.793	2.781	0.38
**7T-T1 TSE-fast**	3.011	3.000	0.90	2.936	3.007	0.13	2.812	2.836	0.13
**7T-T1 TSE-HR**	2.978	3.121	0.01	2.993	3.047	0.12	2.832	2.839	0.53

**Table 4:** Comparison of horizontal fractal dimensions between radiography and different MRI T1 sequences in the “patient” group.

	Comparison 1 (Sample Size, *n* = 23)			Comparison 2 (Sample Size, *n* = 18)	
	Radiography (Reference)	3T-T1 TSE (*p*-Value)	7T-T1 TSE-HighRes (*p*-Value)	Radiography (Reference)	7T-T1 TSE-Fast (*p*-Value)
**Low Resolution** **1.8–2.7 mm**	3.004	3.139 (0.03)	3.201 (0.0002)	2.986	3.069 (0.13)
**Medium Resolution** **0.9–1.8 mm**	2.988	2.971 (0.56)	3.01 (0.46)	3.001	2.952 (0.20)
**High Resolution** **0.0–0.9 mm**	2.7	2.632 (<0.0001)	2.732 (0.01)	2.7	2.691 (0.59)

**Table 5:** Comparison of vertical fractal dimensions between radiography and different MRI T1 sequences in the “patient” group.

	Comparison 1 (Sample Size, *n* = 23)			Comparison 2 (Sample Size, *n* = 18)	
	Radiography (Reference)	3T-T1 TSE (*p*-Value)	7T-T1 TSE-HighRes (*p*-Value)	Radiography (Reference)	7T-T1 TSE-Fast (*p*-Value)
**Low Resolution** **1.8–2.7 mm**	3.107	3.043 (0.37)	2.996 (0.14)	3.129	3.047 (0.19)
**Medium Resolution** **0.9–1.8 mm**	2.982	2.952 (0.42)	3.003 (0.59)	2.977	2.942 (0.38)
**High Resolution** **0.0–0.9 mm**	2.837	2.79 (0.0005)	2.831 (0.57)	2.833	2.81 (0.07)

## Data Availability

Not applicable.
